# The plasticity of HTLV-1 infected CD4+CD25+CCR4+ T-cells through HTLV-1 tax in HAM/TSP

**DOI:** 10.1186/1742-4690-8-S1-A104

**Published:** 2011-06-06

**Authors:** Natsumi Araya, Tomoo Sato, Atae Utsunomiya, Hitoshi Ando, Naoko Yagishita, Mari Kannagi, Tatsufumi Nakamura, Yuetsu Tanaka, Steven Jacobson, Yoshihisa Yamano

**Affiliations:** 1 Department of Molecular Medical Science, Institute of Medical Science, St. Marianna University School of medicine, Kawasaki, Japan; 2 Department of Hematology, Imamura Bun-in Hospital, Kagoshima, Japan; 3 Department of Immunotherapeutics, Tokyo Medical and Dental University, Graduate School, Tokyo, Japan; 4 Department of Molecular Microbiology and Immunology, Graduate School of Biomedical Sciences, Nagasaki University, Nagasaki, Japan; 5 Department of Immunology, Graduate School of Medicine, University of the Ryukyus, Okinawa, Japan; 6 Viral Immunology Section, Neuroimmunology Branch, National Institute of Neurological Disorders and Stroke, National Institutes of Health, Bethesda, Maryland, USA

## 

Recently, it has become increasingly clear that some committed effecter and regulatory T (Treg) cells are not stable, and the plasticity of committed T-cells may be related to autoimmunity and inflammatory disease. However, the environmental (extrinsic) molecules that allow for plasticity have not yet been clearly understood.

In Human T-lymphotropic virus type 1 (HTLV-1) associated myelopathy /tropical spastic paraparesis (HAM/TSP), the pathogenesis is known as HTLV-1 infected CD4+ T-cells triggered hyper immune response, which leads to chronic inflammation of the central nervous system. In our previous study, we demonstrated that the majority of CD4+CD25+CCR4+ T-cells were infected with HTLV-1 and that this T-cell subset was increased in HAM/TSP. Although CD4+CD25+CCR4+ T-cells of healthy condition include suppressive T cell subsets such as Treg and Th2, this T-cell subset becomes Th1-like cells with overproduction of IFN-γ in HAM/TSP patients (PLoS ONE 2009). Since HTLV-1 tax is known to up-regulate the expression of several proinflammatory cytokines, and importantly, the level of HTLV-1 tax mRNA expression is reported to correlate with disease severity in HAM/TSP patients, we hypothesize that HTLV-1 tax may convert HTLV-1 infected T-cells into abnormal IFN-γ producing Th1-like T-cells in HAM/TSP. In this study, we present the molecular mechanisms underlying the plasticity of HTLV-1 infected CD4+CD25+CCR4+ T-cells through HTLV-1 tax.

